# Acoustic Vulnerability, Hydraulic Capacitance, and Xylem Anatomy Determine Drought Response of Small Grain Cereals

**DOI:** 10.3389/fpls.2021.599824

**Published:** 2021-05-25

**Authors:** Szanne Degraeve, Niels J. F. De Baerdemaeker, Maarten Ameye, Olivier Leroux, Geert Jozej Willem Haesaert, Kathy Steppe

**Affiliations:** ^1^Department of Plants and Crops, Faculty of Bioscience Engineering, Ghent University, Ghent, Belgium; ^2^Laboratory of Plant Ecology, Department of Plants and Crops, Faculty of Bioscience Engineering, Ghent University, Ghent, Belgium; ^3^Laboratory of Applied Mycology and Phenomics, Department of Plants and Crops, Faculty of Bioscience Engineering, Ghent University, Ghent, Belgium; ^4^Department of Biology, Faculty of Sciences, Ghent University, Ghent, Belgium

**Keywords:** acoustic emission, drought stress, hydraulic capacitance, small grain cereals, vulnerability curve, xylem anatomy, xylem embolism

## Abstract

Selection of high-yielding traits in cereal plants led to a continuous increase in productivity. However, less effort was made to select on adaptive traits, favorable in adverse and harsh environments. Under current climate change conditions and the knowledge that cereals are staple foods for people worldwide, it is highly important to shift focus to the selection of traits related to drought tolerance, and to evaluate new tools for efficient selection. Here, we explore the possibility to use vulnerability to drought-induced xylem embolism of wheat cultivars Excalibur and Hartog (*Triticum aestivum* L.), rye cultivar Duiker Max (*Secale cereale* L.), and triticale cultivars Dublet and US2014 (*x Triticosecale* Wittmack) as a proxy for their drought tolerance. Multiple techniques were combined to underpin this hypothesis. During bench-top dehydration experiments, acoustic emissions (AEs) produced by formation of air emboli were detected, and hydraulic capacitances quantified. By only looking at the AE_50_ values, one would classify wheat cultivar Excalibur as most tolerant and triticale cultivar Dublet as most vulnerable to drought-induced xylem embolism, though Dublet had significantly higher hydraulic capacitances, which are essential in terms of internal water storage to temporarily buffer or delay water shortage. In addition, xylem anatomical traits revealed that both cultivars have a contrasting trade-off between hydraulic safety and efficiency. This paper emphasizes the importance of including a cultivar’s hydraulic capacitance when evaluating its drought response and vulnerability to drought-induced xylem embolism, instead of relying on the AE_50_ as the one parameter.

## Introduction

Cereals, in particular rice, wheat, and maize, provide a major source of carbohydrates, proteins, vitamins, and minerals, and constitute more than 50% of the calories of the global population’s diet (McKevith, 2004; Laskowski et al., 2019). During the green revolution, the selection of high-yielding traits in cereal plants led to a continuous increase in productivity. However, historically less emphasis was given to adaptive traits, which are favorable in adverse and harsh environments, leaving crops at risk of failure under current climate change conditions (Choudhary et al., 2019). This impacts crop production, food access, price stability, and therefore food security in several regions of the world (Porter et al., 2014; FAO et al., 2018). Without the adaptation of current agronomic practices, this is expected to exacerbate as temperatures increase and climate extremes (e.g., droughts, floods, and storms) prevail more frequently and with higher intensity (IPCC, 2014). Drought in particular causes already more than 80% of the total climate-related damage and losses in agriculture. So, due to their importance, it is crucial to shift focus to the development of cereals resilient to current and future arid conditions, and to evaluate new tools for efficient selection. Different traits associated to drought tolerance exist in cereals. With a focus on plant survival, osmotic adjustment is an important mechanism by which plants postpone dehydration stress in dry environments (Farooq et al., 2009; Blum, 2014). Sustained turgor governed by active accumulation of solutes in the cytoplasm helps maintaining cell elongation and expansion and therefore plant growth and development. Cereals could also benefit from the ability to optimize root distribution and architecture dependent on soil water distribution. This guarantees a continuous water flow to the aboveground plant parts (Tricker et al., 2018). Vadez (2014) highlights the importance of water availability during the grain filling stage. Water extracted from the soil during grain filling almost fully contributes to grain growth since there is only limited vegetative growth after anthesis. In this regard, traits regulating the plant’s water use efficiency, e.g., stomatal conductance, canopy development, and transpiration, are the most promising targets for breeding resilient new varieties (Vadez et al., 2013). However, when soil water supply becomes limiting during grain filling, drought tolerance will be dependent on the initial concentration of remobilizable carbohydrates in the stem and the efficiency of remobilization to the ear (Blum, 1998). Water soluble carbohydrates (WSCs) accumulated in the stem prior to flowering, and shortly after flowering, usually contribute for 10–20% of the grain yield, but under terminal dry conditions, they potentially account for 40–60% of the grain weight. Evidently, the ability of plants to regulate their water status requires a fully functional water transport system. Xylem is the tissue specialized in the long-distance transport of water, and according to the cohesion-tension theory (Dixon and Joly, 1895; Tyree and Zimmermann, 2002; Venturas et al., 2017), water is passively transported through the plant’s vascular system. This passive transport originates in the evaporation of water in the substomatal cavities of the leaves (i.e., transpiration), resulting in a negative water potential, enabling the flow of water upward from the roots. However, an increasing evaporative demand and/or drying soil will result in decreasing water potential (more negative values) in the xylem conduits and may cause air nanobubbles entering the xylem vessels. When the nanobubbles coalesce, expand, and fill the entire conduit (Schenk et al., 2015), the water transport system is locally interrupted. Water from these embolized vessels may still contribute to the transpiration stream as water recedes into adjacent vessels via intervessel pit membranes (Venturas et al., 2017). These pit membranes additionally prevent the spread of air emboli throughout the vascular system by exerting strong capillary forces (Choat et al., 2008; Venturas et al., 2017). However, when negative pressure in the xylem vessels outpaces these capillary forces, nanobubbles can “seed” from one adjacent vessel to another, forming embolisms in the plant’s entire hydraulic system and eventually leaving the plant to dehydrate to lethal levels (Tyree and Zimmermann, 2002).

The vulnerability of cereals to drought-induced xylem embolism could serve as a key trait for its drought tolerance (Choat et al., 2012). Assessing a species hydraulic safety is typically done by constructing a vulnerability curve (VC), in which the percentage loss of xylem hydraulic conductivity (PLC, %) is plotted against decreasing xylem water potential (Ψ_*xylem*_, MPa) as indicator of increasing drought stress. Different methods to determine xylem hydraulic conductivity have been explored and reviewed (Cochard et al., 2013), including detection of acoustic emissions (AEs) produced by the formation of air bubbles (De Roo et al., 2016; Vergeynst et al., 2016), direct observations of the presence of air bubbles in the xylem (Choat et al., 2016; Brodribb et al., 2017; Johnson et al., 2018; Corso et al., 2020), and hydraulic detection of the decrease in xylem transport efficiency (Nolf et al., 2017). The water potential at which 50% loss of hydraulic conductivity occurs (P_50_ or AE_50_, depending on the method used to determine hydraulic conductivity) is commonly used to interpret a species vulnerability. Such hydraulic parameters have been determined in several woody plants (Choat et al., 2012; Nolf et al., 2015; Epila et al., 2017), but despite their economic importance, few studies exist in cereal crops (Stiller et al., 2003; Gleason et al., 2017; Johnson et al., 2018; Corso et al., 2020). And if changes in hydraulic conductivity are determined, quantification of hydraulic capacitance, i.e., the amount of water released (ΔW; kg) for a given change in xylem water potential (ΔΨ; MPa) per unit of tissue volume (V; m^3^) (Tyree and Ewers, 1991), is often lacking (Vergeynst et al., 2015a).

In this study, we assess the potential of using vulnerability to drought-induced xylem embolism as a proxy for drought resistance of different small grain cereals. Multiple techniques are therefore combined to apprehend the different aspects of xylem vulnerability to drought. (1) Through continuous measurements of AEs during bench-top dehydration, AE_50_ values are quantified and vulnerability to drought-induced xylem embolism assessed. (2) Hydraulic capacitance and capacitive water release during dehydration are derived from desorption curves (DC) after continuously weighing of samples. To corroborate these results, (3) xylem anatomical traits linked with a species’ individual trade-off between hydraulic safety and efficiency (Hacke and Sperry, 2001; Sperry et al., 2008) are determined. This set of techniques is used to assess vulnerability of wheat (*Triticum aestivum* L.), triticale (*x Triticosecale* Wittmack), and rye (*Secale cereale* L.) to drought-induced xylem embolism. Wheat is included because of its economic importance and high productivity, rye because of its robustness to all kinds of abiotic stresses, and triticale, as a cross between the former ones, is believed to combine the best of both. In this regard, we would expect AE_50_ values of rye and triticale to be highest.

## Materials and Methods

### Plant Materials and Sampling Procedure

In this study, two spring wheat (*T. aestivum* L.) cultivars, Excalibur and Hartog, two spring triticale (*x Triticosecale* Wittmack) cultivars, Dublet and US2014, and one spring rye (*S. cereale* L.) cultivar, Duiker Max, were used (Table 1). For each cultivar, three 4-L pots, filled with potting soil (Jardino Basic, BVB Substrates, Netherlands), contained 10 seedlings. During the entire growing cycle, seedlings were well watered and grown in a climate chamber at 20°C and 12/12 h light/dark conditions (GRO-LUX SHP-TS 400W E40 SLV, Sylvania, Budapest, Hungary). Plants were fertilized with an NPK fertilizer (6/6/7 ratio) at the start of the elongation phase (Zadok Scale, Z30; Zadoks et al., 1974) and when the flag leaf appeared (Z40).

**TABLE 1:** Different cultivars of wheat, triticale, and rye used in this study.

Cultivar	Species	Origin	Breeder	Source
Excalibur	*Triticum aestivum* L.	Australia	University of Adelaide, Roseworthy and South Australian Research and Development Institute	CIMMYT
Hartog	*Triticum aestivum* L.	Australia	Queensland Wheat Research Institute	CIMMYT
Dublet	x *Triticosecale* Wittmack	Poland	Danko	Danko
US2014	x *Triticosecale* Wittmack	South Africa	Stellenbosch University	Stellenbosch University
Duiker Max	*Secale cereale* L.	South Africa	Stellenbosch University	Stellenbosch University

**FIGURE 1:**
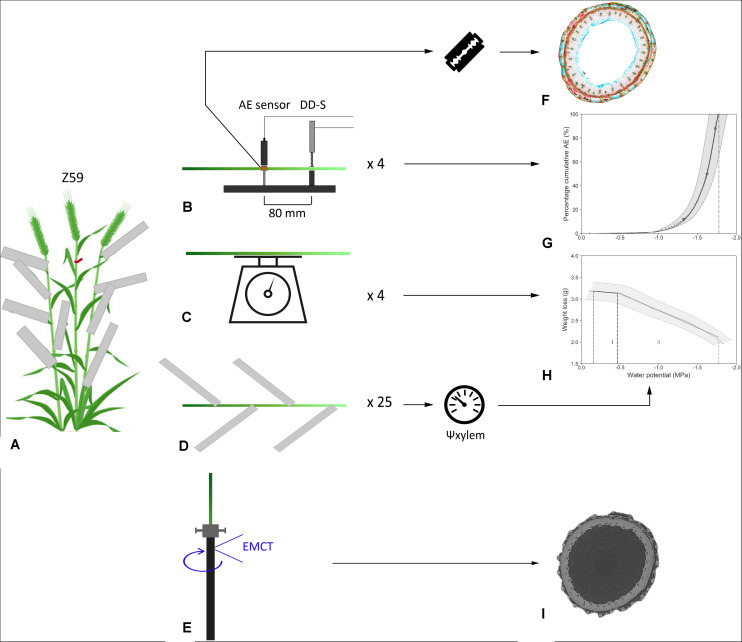
Illustration of the workflow for **(A)** sampling and **(B–D)** measurements during bench dehydration, processing the data into **(G)** vulnerability and **(H)** desorption curves, **(F)** preparation of stem cross-sections, and **(E,I)** experimental setup of *T. aestivum* cultivar Excalibur in the EMCT.

Plant material was collected at anthesis with the ear fully emerged (Z59). The day prior to the start of the experiment, eight uniform main shoots were tagged for the determination of VC_AE_ and hydraulic capacitances. At the same time, leaves of the remaining shoots were wrapped in aluminum foil to ensure equilibrium between leaf and stem water potential (Begg and Turner, 1970) at the start of the experiment (Figure 1A). The day of the experiment, selected shoots were excised just above the root zone while under water to avoid artificial embolism formation. The ends of the cut stems were covered with wet paper towel to prevent dehydration during installation. In addition, the cut stems were stripped of the ear and all leaves and wounds were covered with petroleum jelly (Vaseline, Unilever) to prevent water evaporating via the wounds. Sampling and installation occurred at room temperature (20°C) and under artificial green light to limit photosynthesis and transpiration.

### Measurements During Bench Dehydration

To develop VC_AE_s, four shoots were mounted in custom-built holders (Figure 1B) which ensured a fixed distance (80 mm) between the broadband point-contact AE sensor (KRNBB_PC, KRN Services, Richland, WA, United States) and the dendrometer (DD-S, Ecomatik, Dachau, Germany). To ensure good acoustic contact, a droplet of vacuum grease (High-Vacuum Grease, Dow Corning, Seneffe, Belgium) was added between the AE sensor and the sample. The pencil lead break test, as described in Vergeynst et al. (2015b), served as validation for the sensor functioning. The AE sensor and dendrometer were placed at the third and second internodes, respectively. Depending on the total length of the shoot, the distance between the end of the shoot and the dendrometer was 18.9 ± 3.5 cm on average and the distance between the end of the shoot and the AE sensor was 26.9 ± 3.5 cm on average. To obtain DCs, mass loss of the other four shoots was recorded continuously using weighing balances (DK 6200 with 0.01 g accuracy, Henk Maas, Veen, Netherlands; Figure 1C). The wet paper towel was removed from both the VC and the DC samples after finishing the experimental set-up. Bench dehydration occurred under laboratory conditions and artificial white light. Readings from balances and dendrometers were registered every minute by custom-built acquisition boards. AE signals were amplified by 35.6 dB (AMP-1BB-J, KRN Services, Richland, WA, United States) in order to acquire waveforms of 7168 samples length at 10 MHz sample rate. Signals were collected via two-channel PCI boards and redirected to the AEwin software (PCI-2, AEwin E4.70, Mistras Group BV, Schiedam, Netherlands). A 20–1000 kHz electronic band-pass filter was applied to only retain waveforms above the noise level of 28 dB_AE_ (Vergeynst et al., 2016).

The remaining shoots, with leaves wrapped in aluminum foil, were also excised under water and left to dehydrate under the same laboratory conditions (Figure 1D). The pressure chamber (PMS Instrument Company, Corvallis, OR, United States) was used to measure xylem water potential (Ψ_xylem_, MPa). The frequency of Ψ_xylem_ readings was guided by the appearance of speed of AE signals, which were monitored in real time with the AEwin software program.

### Processing Acoustic Emission Data Into Vulnerability Curves

The AE signals were translated into meaningful VC_AE_s (Vergeynst et al., 2016; De Baerdemaeker et al.,2019a,b). AE signals per sample were cumulated and averaged over 5 min over the entire dehydration period. By calculating the first derivative over a time interval of 15 min, an AE activity curve was drafted. According to Vergeynst et al. (2016), the endpoint of the VC_AE_ (AE_100_) was determined as the point at which the AE activity, following the AE activity peak, decreases most strongly, which mathematically corresponds to the local maximum of the third derivative. The time interval to calculate the third derivative is dependent on the duration of the bench-top dehydration period, the studied species, and the dehydration conditions (Vergeynst et al., 2016). For the species studied in this experiment, a time interval of 2–3 h was sufficient in order to obtain consistent results. Cumulative AE was rescaled from zero to the defined endpoint to obtain meaningful PLC (%). Values corresponding with the onset of xylem embolism (AE_12_), 50% embolism-related AE (AE_50_), 88% embolism-related AE (AE_88_), and full embolism (AE_100_) can be determined from the VC_AE_ (Figure 1G). Using a segmented-linear regression relationship between relative radial xylem shrinkage (Δd/d_i_, μm.mm^–1^), measured with the dendrometer, and point measurements of Ψ_xylem_ (MPa), a continuous *x*-axis (Ψ_xylem_, MPa) for each VC_AE_ was realized.

### Validation of the Acoustic Emission Measurements Using X-Ray Microcomputed Tomography

For *T. aestivum* cultivar Excalibur, one shoot in Z59 was excised under water, stripped of ear and leaves, and mounted on the rotation stage of the environmental microCT scanner (EMCT) (Dierick et al., 2014), a custom-built CT scanner at the Center for X-ray CT scanning of Ghent University (UGCT), Belgium. This scanner rotates around its mounting stage instead of having a rotating stage and allows several subsequent scans because a full rotation brings the scanner back to its starting position. The maximum resolution obtained with the EMCT is 5 μm.

The shoot was fixed in a custom-built holder designed to prevent the shoot from vibrating during scanning, which would make it impossible to visualize the xylem vessels. The tube enclosing the shoot was made of carbon fiber, allowing easy penetration of X-rays (Figure 1E). During the experiment, as the shoot was dehydrating, we noticed shrinkage. As a result, the shoot was no longer tightly enclosed by the tube and started vibrating. Accordingly, Teflon was applied just above and below the scanning position, which was 180 mm from the end of the shoot (at the second internode) to eliminate vibration.

The shoot was scanned hourly from 11 a.m. until 6 p.m. and from 9 a.m. until 1 p.m. the next day. The tube voltage was 60 kV, the tube power was 6 W, and no additional filter was applied. A rotation lasted 9 min, with 1801 projections taken per rotation. The approximated voxel size was 4.99 μm. Between each run, the shoot was removed from the carbon tube and left at the benchtop to dehydrate. Afterward, images were reconstructed (Figure 1I), using the Octopus reconstruction software package.

### Desorption Curves and Hydraulic Capacitances

Mass loss, registered by the balances during dehydration, is a surrogate for overall change in water content (ΔWC, g) and was plotted against the continuous Ψ_xylem_-axis (MPa, Figure 1H). The DC shows two interesting regions, separated by two defined breakpoints, calculated via the segmented R package (Muggeo, 2008). A first and a second breakpoint indicate the start and end of phase I, or the elastic shrinkage phase. Starting from the second breakpoint till AE_100_ is known as inelastic shrinkage phase (xylem embolism) or phase II (Vergeynst et al., 2015a). According to Vergeynst et al. (2015a), hydraulic capacitance (C, g MPa^–1^) for both phases (C_el_ and C_inel_, respectively) was calculated as the slope of the linear regression between mass loss and Ψ_xylem_.

### Microscopic Analysis

Segments of the third internode of approximately 3 cm, including the position where the AE sensor was positioned (26.9 ± 3.5 cm from the end of the shoot), were collected from all the shoots used for VC_AE_ measurements at the end of the dehydration period (Figure 1B, red square). These samples were stored in tubes containing a 20:40:40 ratio of 70% ethanol, glycerol, and distilled water. In addition, after finishing EMCT scanning, a segment of the stem at the scanning position was also collected and stored. After a pre-treatment in 2% (v/v) hydrofluoric acid and 0.5% (v/v) sulphuric acid for 48 h, samples were rinsed thoroughly in demineralized water and embedded in 8% (w/v) agarose. Agarose blocks containing the samples were glued onto the vibratome stage. Cross-sections of 70 μm thickness were produced with a vibration microtome (HM 650V, Thermo Scientific, Germany) and were stained with 0.5% (w/v) astra blue, 0.5% (w/v) chrysoidine, and 0.5% (w/v) acridine red to distinguish lignified (red) from cellulose (blue) cells (Figure 1F). Sections were finally mounted in Euparal (Carl Roth, Germany) and imaged using a Nikon Ni-U microscope equipped with a Nikon DS-Fi1c camera. The open source software Fiji was used for image analysis.

**FIGURE 2:**
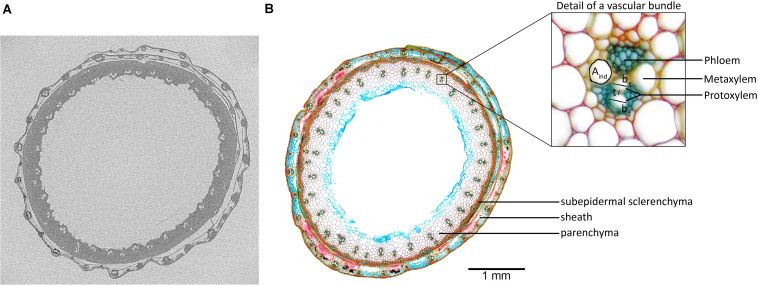
**(A)** Reconstruction of the EMCT image of Excalibur, taken 26 h after onset of dehydration. From this exact scanning position, **(B)** a 70-μm-thick cross-section was made and stained with 0.5% (w/v) astra blue, 0.5% (w/v) chrysoidine, and 0.5% (w/v) acridine red. Individual vessel area (Aind), wall span (b), and wall thickness (t) are indicated on the detailed magnification of the vascular bundle.

Both protoxylem and metaxylem were included in the analysis. Individual vessel areas (A_ind_, μm^2^; Figure 2B detail) and total vessel area (A_total_, μm^2^) were determined directly from the images and, assuming a circular vessel shape, individual vessel diameters (d, μm) were calculated. Hydraulic diameter (d_h_, μm) was calculated as described by Steppe and Lemeur (2007):

$${\text{d}}_{\text{h}}=\sqrt[{4}]{\frac{1}{\text{n}}\,\sum_{i=1}^{\text{n}}{\text{d}}_{\text{i}}^{4}}$$

Conduit wall reinforcement (CWR) was calculated by determining the double wall thickness (t) to conduit wall span (b) ratio (Figure 2B detail) as described in Hacke et al. (2001):

$$\mathrm{CWR}={\left(\text{t}\,/\,\text{b}\right)}_{\text{h}}^{2}$$

Vessel grouping index (V_g_) was determined as the ratio of total number of vessels to total number of vessel groupings. The above were calculated separately for both stem and leaf sheath. In addition, area xylem parenchyma in the stem was derived from the images and allowed calculation of ratio xylem parenchyma to total stem area (%_Xylem Parenchyma_).

## Results

### Acoustic Emissions by Drought-Induced Embolism and Hydraulic Capacitance

**FIGURE 3:**
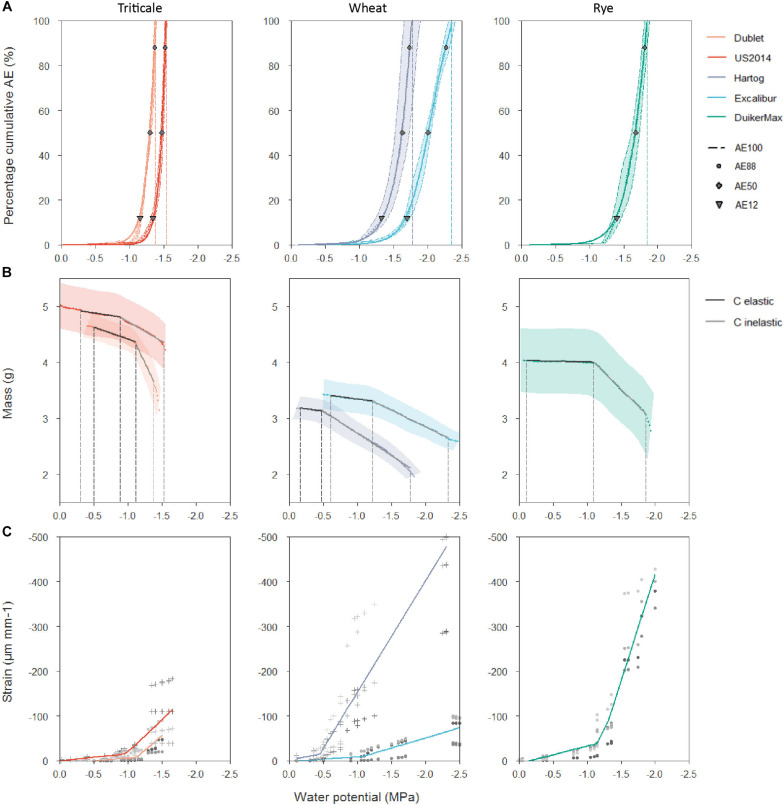
**(A)** Averaged vulnerability curves (VCAEs) with standard error bands of triticale cultivars Dublet and US2014, wheat cultivars Hartog and Excalibur and rye cultivar Duiker Max. AE_12_, AE_50_, AE_88_, and AE_100_ are indicated. **(B)** Averaged desorption curves (DCs) with standard error bands for the same cultivars. Elastic and inelastic shrinkage are delimited by vertical dashed lines, and the corresponding hydraulic capacitances (C, g MPa^–1^) of both phases are calculated as the slope of the linear regressions. **(C)** General stress–strain relationship for all five cultivars. The different shade colors for or + indicate the different shoots for each cultivar.

Acoustic emission measurements were taken to determine a cultivar’s vulnerability to drought-induced xylem embolism (Figure 3A). Generally, AE_50_ values are used as a parameter to quantify vulnerability, with a more negative value indicating a more tolerant species. Acoustic vulnerability analysis showed the most negative AE_50_ value for wheat cultivar Excalibur (Table 2). Its AE_50_ value of −2.00 MPa is significantly more negative than the other cultivars tested. Triticale cultivars Dublet and US2014 had the highest AE_50_ values of −1.30 and −1.47 MPa, respectively. Values corresponding with the onset of xylem embolism (AE_12_), 50% embolism-related AE (AE_50_), 88% embolism-related AE (AE_88_), and full embolism (AE_100_) are indicated in Figure 3A. VC_AE_s show that only a small decrease in xylem pressure is required to shift from onset of cavitation to full embolism, for all cultivars. For wheat cultivar Excalibur, xylem pressure ranged from −1.70 ± 0.07 MPa (AE_12_) to −2.35 ± 0.09 MPa (AE_100_) and for triticale cultivar Dublet, xylem pressure ranged from −1.16 ± 0.03 MPa (AE_12_) to −1.38 ± 0.03 MPa (AE_100_). The timespan necessary to reach full embolism (t_100%_, hours, Table 2) was longest for Duiker Max and Dublet (32.1 ± 5.9 and 32.0 ± 5.6 h, respectively) and shortest for Excalibur (21.0 ± 0.6 h). Nolf et al. (2015) hypothesized that the highest acoustic activity should occur when most embolism is forming within a narrow range of Ψ_xylem_ and therefore should be correlated to the AE_50_ value. We also found a positive linear correlation (*R*^2^ = 0.928; *AE*_*maxactivity*_ = 0.909*AE_50_-0.174) between the species’ AE_50_ values and the Ψ_xylem_ at maximum AE activity (AE_max activity_).

**TABLE 2:** Water potential at which 12, 50, 88, and 100% loss of hydraulic conductivity occurs (AE_12_, AE_50_, AE_88_, and AE_100_), dehydration time till complete embolism formation (t_100%_), elastic capacitance (C_el_), and inelastic capacitance (C_inel_) for the different cultivars used in this study.

	Dublet	US2014	Hartog	Excalibur	Duiker Max
AE_12_ (MPa)	−1.16 ±0.03^a^	−1.34 ±0.05^a^	−1.32 ±0.18^a^	−1.70 ±0.07^b^	−1.40 ±0.13^a^
AE_50_ (MPa)	−1.30 ±0.03^a^	−1.47 ±0.04^a,b^	−1.62 ±0.21^b^	−2.00 ±0.07^*c*^	−1.67 ±0.12^b^
AE_88_ (MPa)	−1.37 ±0.03^a^	−1.52 ±0.03^a,b^	−1.73 ±0.22^b,c^	−2.27 ±0.09^*d*^	−1.81 ±0.08^*c*^
AE_100_ (MPa)	−1.38 ±0.03^a^	−1.54 ±0.03^a,b^	−1.78 ±0.24^b,c^	−2.35 ±0.09^*d*^	−1.84 ±0.08^*c*^
t_100%_ (hours)	32.02 ±5.63^a,b^	27.34 ±3.90^a,b,c^	22.58 ±1.96^b,c^	20.95 ±0.63^*c*^	32.14 ±5.90^a^
C_el_ (g MPa^–1^)	0.42 ±0.10^a^	0.20 ±0.04^b^	0.15 ±0.08^b^	0.17 ±0.07^b^	0.04 ±0.07^b^
C_inel_ (g MPa^–1^)	2.48 ±0.72^a^	0.68 ±0.12^b^	0.82 ±0.34^b^	0.57 ±0.09^b^	1.19 ±0.32^b^

*Values are mean ± standard deviation. Letters (a, b, c, and d) indicate significant differences after a one-way ANOVA and Tukey test (*p* = 0.001).*

Continuously weighing of the stem samples led to quantification of the hydraulic capacitance and capacitive water release potential. The capacity of vascular plants to store water in its tissues and release of this water in the event that stress strains the hydraulic integrity could be a significant drought tolerance strategy. Hydraulic elastic (originating from living tissues) and inelastic (originating from xylem embolism) capacitances (Table 2) were significantly higher for triticale cultivar Dublet (C_el_: 0.42 ± 0.10 g MPa^–1^ and C_inel_: 2.48 ± 0.72 g MPa^–1^), compared to the other cultivars in this experiment. Figure 3B shows that Dublet has the greatest capacitive water release per decreasing unit of xylem water potential. The other cultivars showed no significant differences, with elastic capacitances ranging from 0.04 ± 0.07 to 0.20 ± 0.04 g MPa^–1^ and inelastic capacitances ranging from 0.57 ± 0.09 to 1.19 ± 0.32 g MPa^–1^.

### Validation of Acoustic Measurements Using X-Ray Microcomputed Tomography

Visual embolism detection on EMCT images closely corresponds to the micrograph of the same scanning position (Figure 2). The cross-section of the stem shows the vascular bundles arranged in two rings (Figure 2B). The ring of small vascular bundles is embedded in subepidermal sclerenchyma, while the second ring is composed of larger vascular bundles enclosed by parenchyma. Around the stem, the leaf sheath is present which also contains larger and smaller vascular bundles in alternating order. According to the EMCT image taken 26 h after onset of dehydration, all 33 large vascular bundles in the stem can be detected, which was not the case for the outer circle of smaller vascular bundles (Figure 2A). The maximum resolution of 5 μm likely explains why these bundles are invisible on the reconstruction image.

**FIGURE 4:**
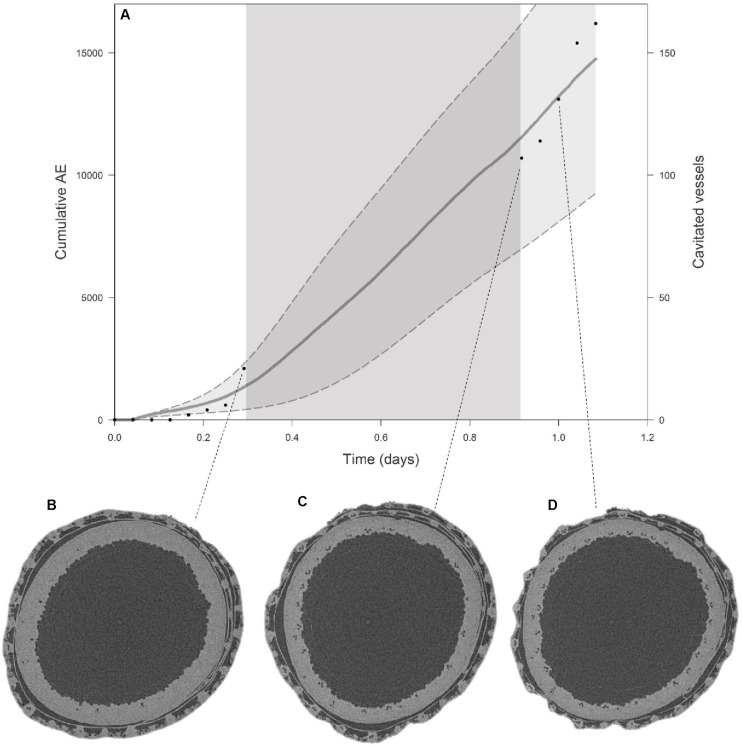
**(A)** Average cumulative AE with standard error bands (gray curve, left *y*-axis) for Excalibur shows a similar pattern to the number of embolized vessels (black dots, right *y*-axis) counted on EMCT images when plotted against time. The dark gray zone in the curve depicts the night hours, when no images could be taken. The EMCT cross-sections for the stem at which 12% **(B)**, 50% **(C)**, and 88% **(D)** of the vessels are embolized are shown. Embolized vessels, filled with air, appear as small black dots on the EMCT cross-sections.

Comparison of visual embolism formation on EMCT images and AE detection for Excalibur (Figure 4A) shows a close correspondence. Seven hours after onset of dehydration, visual embolism detection is slightly higher, but still within the standard error margins of the mean cumulative AE curve. In the morning, when scanning started again, visual embolism detection was at the same level as the mean cumulative AE curve. Figures 4B–D show EMCT images of Excalibur with, respectively, 12, 50, and 88% of the vessels embolized.

### Xylem Microscopic Analysis

Results from stem anatomy (Table 3) revealed that triticale cultivar Dublet had significantly higher A_ind_ (771 ± 120 μm^2^) and d_h_ (34.8 ± 2.9 μm) compared to Excalibur and Duiker Max. Its average total stem area (A_stem_) was also larger (5.04 ± 1.13 mm^2^), though the difference between cultivars was not significant. V_g_ for Dublet (1.20 ± 0.035) was significantly higher than for Hartog (1.11 ± 0.026). On the other hand, Hartog had the highest CWR (0.0386 ± 0.0204) and the greatest area of stem occupied by vessels (2.61 ± 0.40%). Wheat cultivar Excalibur and rye cultivar Duiker Max had lowest values for A_ind_ (424 ± 103 and 457 ± 366 μm^2^, respectively) and d_h_ (24.6 ± 3.2 and 26.6 ± 1.0 μm, respectively). The number of vessels ranged from 149 ± 9 for Duiker Max to 198 ± 20 for Excalibur, but differences between cultivars were not significant. The percentage of stem occupied by xylem parenchyma was greatest in Excalibur and smallest for triticale cultivars Dublet and US2014, although the difference between cultivars was not significant.

**TABLE 3:** Stem anatomical parameters individual vessel area (A_ind_), total vessel area (A_total_), total stem area (A_stem_), ratio xylem parenchyma to total stem area (%_Xylem Parenchyma_), hydraulic diameter (d_h_), number of vessels (N_vessel_), vessel grouping index (V_g_), and conduit wall reinforcement (CWR) for the different cultivars in this study.

	Dublet	US2014	Hartog	Excalibur	Duiker Max
A_ind_ (μm^2^)	771 ±120^a^	662 ±102^a,b^	594 ±185^a,b^	424 ±103^b^	457 ±366^b^
A_total_ (mm^2^)	0.126 ±0.030^a^	0.119 ±0.011^a^	0.090 ±0.029^a,b^	0.086 ±0.028^a,b^	0.068 ±0.008^b^
A_stem_ (mm^2^)	5.04 ±1.13^a^	4.67 ±0.18^a^	3.38 ±0.82^a^	4.27 ±1.09^a^	3.25 ±0.39^a^
%_Xylem Parenchyma_*	2.19 ±0.44 ^a^	2.13 ±0.18^a^	3.24 ±1.11^a^	4.39 ±1.18^a^	3.92 ±1.76^a^
d_h_ (μm)	34.8 ±2.9^a^	31.2 ±2.1^a,b^	29.1 ±5.0^a,b^	24.6 ±3.2^b^	26.6 ±1.0^b^
N_vessel_*	163 ±32^a^	182 ±21^a^	150 ±9^a^	198 ±20^a^	149 ±9^a^
V_g_	1.20 ±0.035^a^	1.13 ±0.042^a,b^	1.11 ±0.026^b^	1.13 ±0.033^a,b^	1.16 ±0.042^a,b^
CWR	0.036 ±0.013^a^	0.033 ±0.006^a^	0.038 ±0.005^a^	0.035 ±0.009^a^	0.022 ±0.002^a^

Values are mean ± standard deviation. Letters represent significant differences after a one-way ANOVA and Tukey test (0.05). For parameters indicated with (*), a Welch correction and Dunnett T3 test (*p* = 0.05) were used.

Anatomical analysis of the leaf sheath showed no significant differences between the cultivars for V_g_ and CWR. Duiker Max showed a significant smaller A_ind_ and d_h_ in the leaf sheath compared to the other cultivars. Duiker Max also had the lowest N_vessel_ and therefore also smallest A_vesseltotal_. When combining stem and leaf sheath anatomical analyses, generally the same pattern was found as with stem anatomical analysis, except for N_vessel_. Results for the leaf sheath and combined anatomical analyses are presented in Supplementary Table 1. Supplementary Figure 1 shows, for every cultivar, a detail of the vascular bundles and the stem anatomy.

## Discussion

Using EMCT as validation technique, we can conclude that the measurements with the AE sensors correctly represent the course of embolism formation for the wheat cultivar Excalibur and by extension also for the other cultivars included in this study.

**FIGURE 5:**
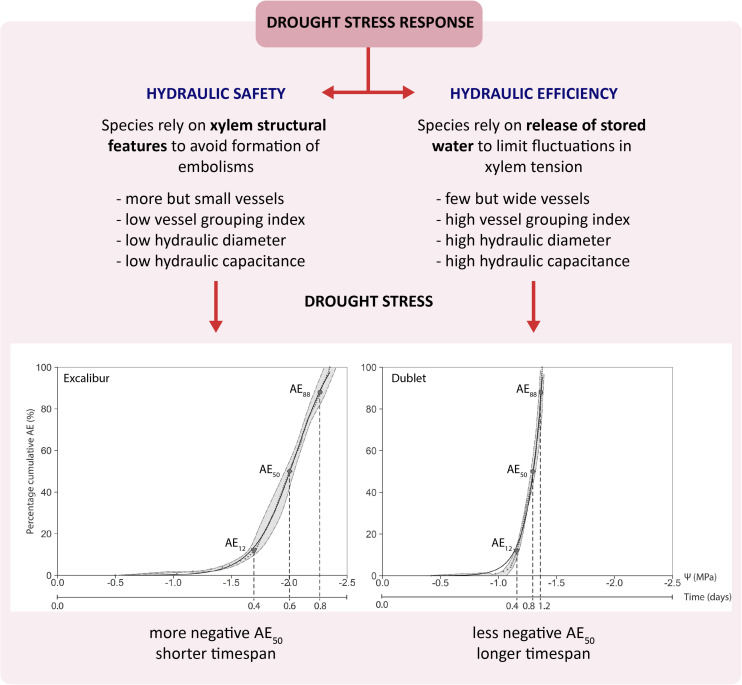
Schematic figure showing the contrasting response of studied cereals to drought stress.

The P_50_ or AE_50_ value is widely seen as relevant parameter to quantify a species’ vulnerability to drought stress (Choat et al., 2012; Anderegg, 2015). The small grain cereals in this study have average AE_50_ values ranging from −1.30 ± 0.03 to −2.00 ± 0.07 MPa (Table 2). The few studies conducted on cereals show similar P_50_ values. Corso et al. (2020) explored the spread of embolism in leaves and stems of wheat (*T. aestivum* var. SY Mattis), using the optical technique and X-ray microCT. They found mean Ψ_leaf_, at which 50% of the vessels were embolized, of −2.21 ± 0.17 MPa using the optical technique and similar values were observed for both leaf and stem with X-ray microCT. A study by Johnson et al. (2018) on wheat (*T. aestivum* var. Heron) used non-invasive imaging and found mean Ψ_leaf_ causing 50% xylem embolism of −2.87 ± 0.52 MPa. Gleason et al. (2017) measured losses of 50% of maximal hydraulic conductivity associated with Ψ_leaf_ values of −1.29 MPa in a greenhouse experiment with maize and Ψ_leaf_ values of −1.15 MP for field plants. In a field trail with up- and lowland rice varieties by Stiller et al. (2003), P_50_ values of −1.6 MPa were measured. When comparing our results with the P_50_ values of a wide range of mainly woody species studied by Choat et al. (2012), we can classify the cereals as being vulnerable to drought-induced embolism. The species in Choat et al. (2012) have P_50_ values ranging from −0.04 to −14.10 MPa and more than 60% have values lower than −2.00 MPa. Comparison of the cultivars in our study shows that Excalibur (*T. aestivum* L.) has the most negative AE_50_ value and could therefore, based on only this criterion, be classified as most tolerant to drought-induced xylem embolism, followed by Duiker Max (*S. cereale* L.), Hartog (*T. aestivum* L.), US2014, and Dublet (x *Triticosecale* Wittmack), respectively. Surprisingly, the timespan necessary to reach full embolism (t_100%_) was longest for Duiker Max and Dublet and shortest for Excalibur (Table 2). When including the DC analysis, Dublet has significantly higher C_el_ and C_inel_ compared to the other cultivars (Table 2). These results point out  that  Dublet    has  a  greater  ability to store    water  in  its living tissue, to use this water to temporarily buffer or delay water shortage and to prolong its life span under drought stress. This clearly points to the importance of including a species’ hydraulic capacitance when evaluating vulnerability to drought-induced xylem embolism. Also Körner (2019) argues that there is no need to attribute a critical role to xylem long distance transport capacity or its failure. Under severe drought, there is no or limited water supply but also limited demand (i.e., demand for water flux approaches zero because of stomatal closure). The remaining capacity of the transport system through the narrow non-embolized tracheids is able to cover this minimal demand. And when completely disconnected from any soil moisture, plants will rely on allocation of their own internal water reserves to the most essential tissues for survival. In this regard, it is assumed that the xylem transport activity could be restored after embolism formation and that living cells provide both water and energy to do so. Secchi and Zwieniecki (2010) found that changes in membrane water permeability during drought and refilling processes could be mediated by members of the PIP1 aquaporin subfamily. Meinzer et al. (2009) hypothesize that P_50_ may have no physiological relevance in the context of stomatal regulation of daily minimum xylem pressure and avoidance of hydraulic failure under non-extreme conditions. Their main results suggest that there is a reliance on different mechanisms which provide hydraulic safety under dynamic conditions. Species with low capacitances appear to rely mostly on xylem structural features to avoid embolism, whereas species with higher capacitances generally rely on transient release of stored water to constrain fluctuations in xylem tension and prevent the formation of air emboli. Analysis of the cultivars’ xylem anatomy (Table 3) also supports the previous findings. Results revealed that Dublet has the highest A_ind_, A_vesseltotal_, A_stem_, d_h_, and V_g_. Significantly lower A_ind_ and d_h_ are found for Excalibur and Duiker Max. These findings relate with a species individual trade-off between hydraulic safety and efficiency (Figure 5). Sperry et al. (2008) defined efficiency as the hydraulic conductivity per cross-sectional area, which is maximized when filled with fewer, though wider and longer vessels, free of internal obstructions. So Dublet’s high individual vessel area (A_ind_) and hydraulic diameter (d_h_) illustrate that this cultivar is characterized by a higher hydraulic efficiency. This allows Dublet to maintain longer under conditions of drought stress. Excalibur, on the other hand, containing more but smaller vessels has a higher hydraulic safety allowing its water potential to become more negative before xylem embolism reaches lethal levels. In addition, Dublet’s high individual vessel area (A_ind_) combined with a higher vessel grouping index (V_g_) makes it easier to allocate water from embolized to intact adjacent xylem vessels (which contributes to C_inel_) and to allocate water stored in living tissue into the transpiration stream (which contributes to C_el_). This contribution to the hydraulic capacity of Dublet explains the extended time span before lethal water potential levels are achieved compared to Excalibur. This contrasting response to drought stress in studied cereals is depicted in Figure 5.

Remarkable is the low C_el_ for Duiker Max, combined with a high C_inel_ and prolonged life span under stress. In this regard, it is worth noting that all cultivars have lower elastic and higher inelastic hydraulic capacitances (Table 2). In addition, the general stress–strain curves, representing the magnitude of elastic shrinkage of the stem in response to drought stress for all five cultivars, show a distinct breakpoint dividing the curves in two phases (Figure 3C). The first part shows a weak shrinkage for the strong decrease in Ψ_xylem_, followed by a strong shrinkage per unit Ψ_xylem_. The breakpoints in the stress–strain curves relate very well with the breakpoints in the DCs, indicating the shift from elastic to inelastic shrinkage. These results indicate that overall the elastic water storage in living tissue of small grain cereals is insufficient to buffer or delay decreases in Ψ_xylem_, while water released from embolized vessels has a more significant contribution toward tempering and delaying xylem tension. These results also support the observations by Hölttä et al. (2009), Vergeynst et al. (2015a), and Epila et al. (2017) who declared that water from embolized vessels is released into the transpiration stream and significantly contributes to a species hydraulic capacitance under drought stress.

**FIGURE 6:**
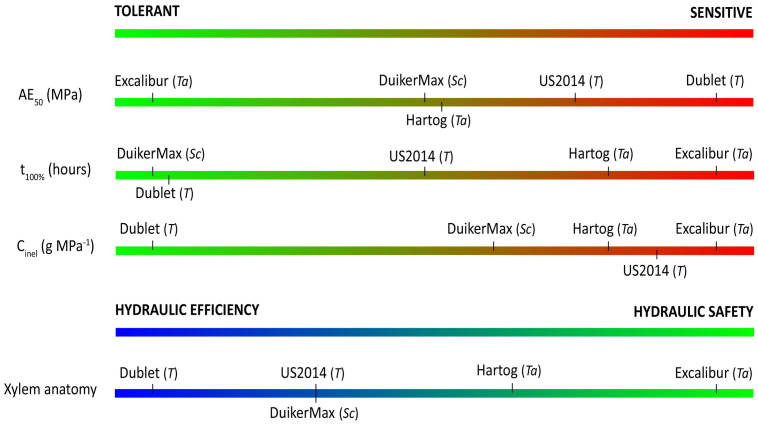
Ranking of cultivars from tolerant to sensitive to drought stress, according to the different parameters explored in this study. Ta, T, and Sc indicate wheat (*T. aestivum* L.), triticale (x *Triticosecale* Wittmack), or rye (*S. cereale* L.).

From the above, it is clear that AE_50_ values do not tell the entire story on vulnerability to drought-induced xylem embolism and that a sole parameter often does not provide a correct assessment of a species’ overall drought stress response. The combination of methods proposed in this paper is necessary to get the full picture–from Ψ_xylem_ at which embolism formation occurs, over hydraulic capacitance and the ability to temporarily buffer or delay water shortage, to a species individual trade-off between hydraulic safety and efficiency—before postulating conclusions on this matter. So, keeping this message in mind and based on the aforementioned parameters, Figure 6 shows our classification of the different cultivars from drought tolerant to drought sensitive per parameter.

## Data Availability Statement

The raw data supporting the conclusions of this article will be made available by the authors, without undue reservation.

## Author Contributions

SD, GH, and KS contributed to the design of the study. SD and NB carried out the experiments. OL provided his expertise on making, staining, and imaging the cross-sections. SD performed the data analysis and wrote the manuscript. All authors contributed to the article and approved the submitted version.

## Conflict of Interest

The authors declare that the research was conducted in the absence of any commercial or financial relationships that could be construed as a potential conflict of interest.

## Supplementary Material

Supplementary Figure 1 Detail of a cross-section from the stem of **(A)** Dublet, **(B)** US2014, **(C)** Hartog, **(D)** Excalibur, and **(E)** Duiker Max. For each cultivar, phloem (ph), metaxylem (m), protoxylem (p), parenchyma (Pa), sclerenchyma (Sc), and lacunae (L) are indicated.
