# Supplementary material for: Acoustic Vulnerability, Hydraulic Capacitance, and Xylem Anatomy Determine Drought Response of Small Grain Cereals
Source: Front Plant Sci. 2021 May 25;12:599824. doi: 10.3389/fpls.2021.599824 (PMC8186553; doi:10.3389/fpls.2021.599824)
Supplement: Supplementary file 1 [file Table_1.DOCX]

Supplementary Material

Table S1: Sheath, stem and combined anatomical analysis with individual vessel area (A_ind_), total vessel area (A_total_), total stem area (A_stem_), ratio xylem parenchyma to total stem area (%_Xylem Parenchyma_), hydraulic diameter (d_h_), number of vessels (N_vessel_), vessel grouping index (V_g_) and conduit wall reinforcement (CWR) for the different cultivars in this study. Values are mean ± standard deviation. Letters represent significant differences after a One-way Anova and Tukey test (0.05). Values indicated with (*) a Welch correction and Dunnett T3 test (p=0.05) was used.

|  | **Cultivar** | **A_ind_ (µm^2^)** | **A_total_ (mm^2^)** | **A_stem_ (mm^2^)** | **%_Xylem Parenchyma_** | **d_h_ (µm)** | **N_vessel_** | **V_g_** | **CWR** |
| --- | --- | --- | --- | --- | --- | --- | --- | --- | --- |
| Sheath | Dublet | 421 ± 65 ^a,b^ | * 0.055 ± 0.055 ^a,b^ |  |  | 24.6 ± 1.8 ^a^ | 69 ± 15 ^a^ | 1.20 ± 0.053 ^a^ | 0.047 ± 0.031 ^a^ |
|  | US2014 | 497 ± 38 ^a^ | * 0.030 ± 0.002 ^a,b^ |  |  | 26.7 ± 0.9 ^a^ | 60 ± 8 ^a,b^ | 1.13 ± 0.039 ^a^ | 0.031 ± 0.005 ^a^ |
|  | Hartog | 484 ± 38 ^a^ | * 0.024 ± 0.002 ^b^ |  |  | 26.2 ± 1.0 ^a^ | 50 ± 7 ^a,b^ | 1.14 ± 0.069 ^a^ | 0.031 ± 0.005 ^a^ |
|  | Excalibur | 493 ± 6 ^a^ | * 0.032 ± 0.002 ^a^ |  |  | 26.3 ± 0.4 ^a^ | 66 ± 5 ^a,b^ | 1.15 ± 0.067 ^a^ | 0.028 ± 0.005 ^a^ |
|  | DuikerMax | 323 ± 58 ^b^ | * 0.014 ± 0.005 ^a,b^ |  |  | 21.4 ± 2.2 ^b^ | 44 ± 13 ^b^ | 1.22 ± 0.030 ^a^ | 0.023 ± 0.006 ^a^ |
| Stem | Dublet | 771 ± 120 ^a^ | 0.126 ± 0.030 ^a^ | 5.04 ± 1.13 ^a^ | * 2.19 ± 0.44 ^a^ | 34.8 ± 2.9 ^a^ | * 163 ± 32 ^a^ | 1.20 ± 0.035 ^a^ | 0.036 ± 0.013 ^a^ |
|  | US2014 | 662 ± 102 ^a,b^ | 0.119 ± 0.011 ^a^ | 4.67 ± 0.18 ^a^ | * 2.13 ± 0.18 ^a^ | 31.2 ± 2.1 ^a,b^ | * 182 ± 21 ^a^ | 1.13 ± 0.042 ^a,b^ | 0.033 ± 0.006 ^a^ |
|  | Hartog | 594 ± 185 ^a,b^ | 0.090 ± 0.029 ^a,b^ | 3.38 ± 0.82 ^a^ | * 3.24 ± 1.11 ^a^ | 29.1 ± 5.0 ^a,b^ | * 150 ± 9 ^a^ | 1.11 ± 0.026 ^b^ | 0.038 ± 0.005 ^a^ |
|  | Excalibur | 424 ± 103 ^b^ | 0.086 ± 0.028 ^a,b^ | 4.27 ± 1.09 ^a^ | * 4.39 ± 1.18 ^a^ | 24.6 ± 3.2 ^b^ | * 198 ± 20 ^a^ | 1.13 ± 0.033 ^a,b^ | 0.035 ± 0.009 ^a^ |
|  | DuikerMax | 457 ± 366 ^b^ | 0.068 ± 0.008 ^b^ | 3.25 ± 0.39 ^a^ | * 3.92 ± 1.76 ^a^ | 26.6 ± 1.0 ^b^ | * 149 ± 9 ^a^ | 1.16 ± 0.042 ^a,b^ | 0.022 ± 0.002 ^a^ |
| Total | Dublet | 668 ± 99 ^a^ | 0.181 ± 0.081 ^a^ |  |  | 32.7 ± 2.7 ^a^ | * 232 ± 47 ^a,b^ | 1.20 ± 0.042 ^a^ | 0.040 ± 0.014 ^a^ |
|  | US2014 | 622 ± 86 ^a,b^ | 0.149 ± 0.010 ^a^ |  |  | 30.3 ± 1.9 ^a,b^ | * 242 ± 29 ^a,b^ | 1.13 ± 0.036 ^a,b^ | 0.032 ± 0.005 ^a^ |
|  | Hartog | 570 ± 146 ^a,b^ | 0.114 ± 0.028 ^a,b^ |  |  | 28.7 ± 4.0 ^a,b^ | * 201 ± 10 ^b^ | 1.11 ± 0.096 ^b^ | 0.036 ± 0.006 ^a^ |
|  | Excalibur | 442 ± 74 ^b^ | 0.118 ± 0.029 ^a,b^ |  |  | 25.2 ± 2.0 ^b^ | * 264 ± 23 ^a^ | 1.14 ± 0.013 ^a,b^ | 0.033 ± 0.007 ^a^ |
|  | DuikerMax | 424 ± 48 ^b^ | 0.082 ± 0.014 ^b^ |  |  | 25.6 ± 1.4 ^b^ | * 193 ± 24 ^a,b^ | 1.18 ± 0.038 ^a,b^ | 0.023 ± 0.003 ^a^ |
