# Supplementary figures and images for: Acoustic Vulnerability, Hydraulic Capacitance, and Xylem Anatomy Determine Drought Response of Small Grain Cereals
Source: Front Plant Sci. 2021 May 25;12:599824. doi: 10.3389/fpls.2021.599824 (PMC8186553; doi:10.3389/fpls.2021.599824)

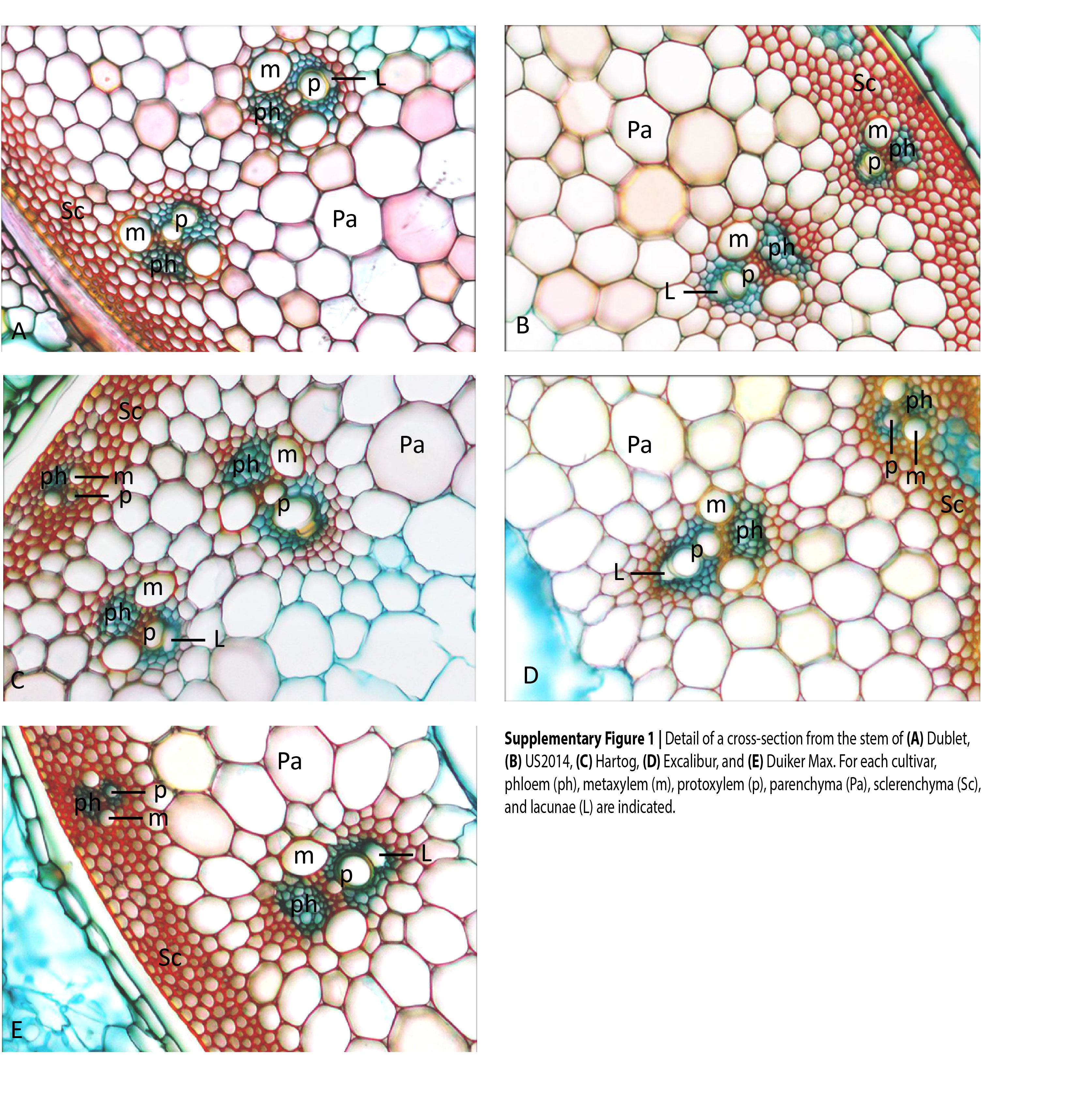

Supplement: Supplementary Figure 1 — Detail of a cross-section from the stem of (A) Dublet, (B) US2014, (C) Hartog, (D) Excalibur, and (E) Duiker Max. For each cultivar, phloem (ph), metaxylem (m), protoxylem (p), parenchyma (Pa), sclerenchyma (Sc), and lacunae (L) are indicated. [file Image_1.jpg]
